# Translational Research in Cystic Fibrosis: From Bench to Beside

**DOI:** 10.3389/fped.2022.881470

**Published:** 2022-05-16

**Authors:** Laura de Castro e Garcia, Lucas Montiel Petry, Pedro Augusto Van Der Sand Germani, Luiza Fernandes Xavier, Paula Barros de Barros, Amanda da Silva Meneses, Laura Menestrino Prestes, Luana Braga Bittencourt, Marina Puerari Pieta, Frederico Friedrich, Leonardo Araújo Pinto

**Affiliations:** Centro Infant, Department of Pediatrics, School of Medicine, Pontifícia Universidade Católica do Rio Grande do Sul, Porto Alegre, Brazil

**Keywords:** treatment, quality of life, cystic fibrosis, CFTR modulators, translational research

## Abstract

Cystic fibrosis is the most common life-limiting recessive genetic disorder in Caucasian populations, characterized by the involvement of exocrine glands, causing multisystemic comorbidities. Since the first descriptions of pancreatic and pulmonary involvement in children, technological development and basic science research have allowed great advances in the diagnosis and treatment of cystic fibrosis. The great search for treatments that acted at the genetic level, despite not having found a cure for this disease, culminated in the creation of CFTR modulators, highly effective medications for certain groups of patients. However, there are still many obstacles behind the treatment of the disease to be discussed, given the wide variety of mutations and phenotypes involved and the difficulty of access that permeate these new therapies around the world.

## Introduction

Cystic fibrosis (CF) is an autosomal recessive genetic disease that affects at least 100,000 people worldwide (1). It is caused by mutations in a gene located on chromosome 7's long arm, which encodes the cystic fibrosis transmembrane conductance regulator (CFTR) protein, a channel responsible for regulating the cotransport of ions across the epithelial cell's membrane (2). This causes the accumulation of exocrine secretions in multiple systems, especially the respiratory, and gastrointestinal systems, causing serious comorbidities (1). Still, lung disease remains the main reason for the high morbidity and mortality in CF. The accumulation of mucus causes vicious cycles of inflammation and recurrent infections, culminating in a progressive reduction in lung function, which can lead to lung transplantation and death. However, in the last decades, relevant advances in the treatment have impacted in patient's life expectancy. Most of the new drugs available for CF treatment were planned and developed from acquired advances on genetics and proteomics.

### Objective

This review aims to provide a summary of developments on diagnostics, proteomics and translational advances including CFTR therapeutics. Recent directions on therapeutics are also discussed in order to present how the novel modulators may impact on life expectancy for almost all individuals with CF.

## Diagnostics

Although there are archaic records of some of these clinical repercussions of CF—described as witchcraft (3)—the characterization of the disease itself only took place in the 1930s, when Dorothy Anderson recognized CF after autopsy studies in malnourished children, giving the disease its “cystic fibrosis of the pancreas” title (4). Over 20 years later, during an intense summer in New York, excessive loss of salt in sweat was discovered in patients with CF, which later culminated in the development of the sweat test, an exam that is now gold standard in the diagnosis of the disease (5, 6). In the last decades, neonatal screening as well and CFTR sequencing have been included as important tool for CF diagnosis. Early initiation of treatment at a specialized referral center by a multidisciplinary team improves clinical outcomes, having a positive impact on patient prognosis (7).

Finally, in 1989, scientists were able to locate and identify the CFTR gene (8–10) contributing to a better understanding of the pathophysiology of CF. Since then, more than 2,000 mutations of the gene have been described (11), among which *F508del* is the most frequent, especially in Caucasian population (12). Such mutations are currently classified into six main classes according to the primary molecular defect, being (I) defect in synthesis, (II) in maturation, (III) in channel, (IV) in conductance, (V) in amount of protein and (VI) in protein stability (13).

### Translational Perspective: From Genetics and Proteomics to the Development of Modulators

The F508del is the most prevalent CF-causing mutation. This mutation leads to CFTR protein misfolding that is arrested by the endoplasmatic reticulum (14). Nevertheless, a small fraction of the mutant protein may evade the quality control checkpoints and reach the membrane.

Other CFTR mutations may impair mRNA and protein expression, function, stability or a combination of these. The classification has historically been evolving according to the gained knowledge (14), and the current scheme is composed of six classes described above. Other systems have also been proposed to take into account the multiple possible variations of CFTR gene (15). However, the classification system in six different classes has been useful in understanding the distinct molecular defects of different CFTR mutations. Also, it became worldwide used to understand the development and effects of pharmacotherapies recently introduced for specific genetic variations.

Mutations in classes I-III are usually associated with a classical and more severe disease, while those in classes IV-VI are related to milder (or atypical) phenotypes. Individuals with CF may nevertheless carry different CFTR mutations on the two alleles, leading to thousands of possible combinations of CF genotypes. Noteworthy, clinical phenotypes and therapeutic responses may differ between individuals carrying the same CF genotypes (16, 17). In fact, several other factors influence disease severity beyond CFTR mutations: gene modifiers, social status, patient's lifestyle, respiratory infections and adherence to therapies (18).

Numerous libraries of compounds have been screened by distinct high-throughput screening (HTS) methods and using several cell models. These experimental approaches contributed to the identification of molecules from different chemical series (19–21).

CFTR modulators enhance or even restore the expression, function, and stability of a defective CFTR channel by distinct mechanisms. CFTR therapeutics have been classified into five main groups depending on their effects on CFTR mutations: potentiators, correctors, stabilizers, read-through agents, and amplifiers (22). To date, four CFTR-directed modulators have reached the market for the treatment of CF patients carrying specific CFTR mutations (23, 24).

### Improvements in the Prognosis in the Era of Modulators

**Figure 1:**
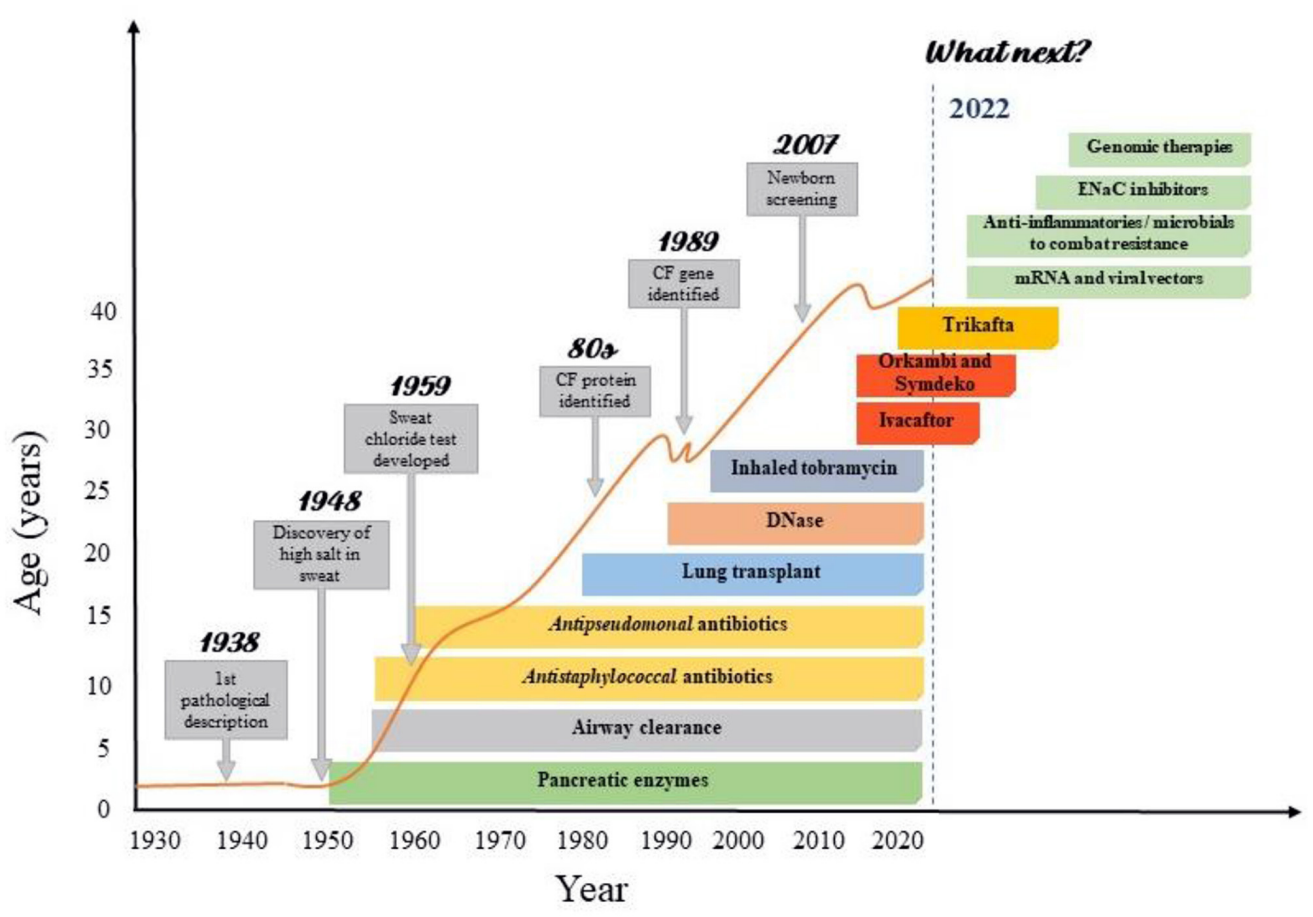
Improvements in cystic fibrosis survival according with the advances CF care. Advances in CF care were resulted from increasing knowledge in basic sciences as genomics and proteomics. The successive advances in the understanding of the pathophysiology, symptoms and treatment achieved by basic research have made the care of patients with cystic fibrosis highly complex, involving protocols for screening, diagnosis and treatment. This has enabled significant gains in life expectancy, especially in recent decades. However, there are still many issues to be resolved in CF, which is why a great development of research with clinical repercussion is expected in the coming years.

Only after clarifying the pathophysiology and identifying the mutations that cause the disease, the arduous search for treatments that acted at the genetic level began (13). Up to the last decade, however, the treatments available for CF were intended only to control the consequence or complications of the disease (Figure 1). Then, in 2011, the publication of a study that demonstrated the effectiveness of the first CFTR modulator (25) started the Era of Modulators, which revolutionized the treatment of CF. The precursor to this series of modulators is Kalydeco® (Ivacaftor), a drug referred to as a CFTR “potentiator”, which was initially intended for use in the treatment of patients with the G551D mutation. Although this discovery was important, the profile of patients eligible for the use of this medication was still very restricted, so that, in subsequent years, the search for drugs that could cover more mutations and produce even more significant results was intensified (Table 1). In the following years, two new therapies were developed: Orkambi® (Ivacaftor/Lumacaftor) and Symdeko® (Tezacaftor/Ivacaftor) (25–27). Lumacaftor is a CFTR “corrector” and its association with Ivacaftor allowed the use of this therapy in homozygous F508del patients, representing a great advance due to the high prevalence of this mutation (28). Tezacaftor is also a CFTR “corrector”, and its combination in Symdeko® has achieved very satisfactory results in patients homozygous for F508del and heterozygous for residual function mutations (26). Even so, none of the modulators produced so far was sufficient to generate results in heterozygous patients with an F508del mutation. It was only 8 years after the production of the first CFTR modulator, that an effective and safe combination of CFTR potentiators and correctors was reached that could be used by patients with a single F508del mutation (23). This combination involves a triple therapy of Elexacaftor, Tezacaftor and Ivacaftor (Trifakta®), which has been approved by the US Food and Drug Administration for patients 12 years and older with at least one F508del mutation, which represents ~90% of the American population with Cystic Fibrosis (29). The results of phase 3 studies were an increase of 10% in FEV1, improved patient-reported quality of life, decrease in sweat chloride value, improvements in BMI, and reduction in the number of pulmonary exacerbations (23, 24, 29).

**Table 1:** Proportion of patients on the CFTR2 platform who are candidates for the use of CFTR modulators as recommended by the US Food and Drug Administration (FDA) and European Medicines Agency (EMA).

**CFTR modulator**	**FDA indication (mutation)**	**EMA indication (mutation)**	% of patients diagnosed with the mutation^*^
Kalydeco® (ivacaftor)	E56K, P67L, R74W, D110E, D110H, R117C, R117H, G178R, E193K, L206W, R347H, R352Q, A455E, S549N, S549R, G551D, G551S, D579G, S945L, S997F, F1052V, K1060T, A1067T, G1069R, R1070Q, R1070W, F1074L, D1152H, G1244E, S1251N, S1255P, D1270N, G1349D	G551D, R117H, G1244E, G1349D, G178R, G551S, S1251N, S1255P, S549N, S549R	8.8%
Orkambi® (ivacaftor/lumacaftor)	F508del homozygous	F508del homozygous	38.2%
Symdeko® (ivacaftor/tezacaftor)	E56K, P67L, R74W, D110E, D110H, R117C, E193K, L206W, R347H, R352Q, A455E, F508del homozygous, D579G, 711 + 3A → G, E831X, S945L, S977F, F1052V, K1060T, A1067T, R1070W, F1074L, D1152H, D1270N, 2789 + 5G → A, 3271-26A → G, 3849 + 10kbC → T	F508del homozygous, P67L, R117C, L206W, R352Q, A455E, D579G, 711 + 3A → G, S945L, S977F, R1070W, D1152H, 2789 + 5G → A, 3272-26A → G, 3849 + 10kbC → T	43.2%
Trikafta® (elexacaftor/tezacaftor/ivacaftor)	At least one F508del mutation or a mutation in the CFTR gene that is responsive based on *in vitro* data^**^	At least one F508del mutation	84.1%

* *Data on the number of patients with specific variants were collected on the CFTR2 data platform (https://cftr2.org/). The percentages are based only on the number of patients who are diagnosed with the mutations cited above. However, age is another important criterion concerning CF treatment. Since we cannot filter data by age on CFTR2, we provided the percentages considering the number of patients of all ages*.

** *Mutations in the CFTR gene that are responsive based on in vitro data: 3141del9, E822K, G1069R, L967S, R117L, S912L, 546insCTA, F191V, G1244E, L997F, R117P, S945L, A46D, F311del, G1249R, L1077P, R170H, S977F, A120T, F311L, G1349D, L1324P, R258G, S1159F, A234D, F508C, H139R, L1335P, R334L, S1159P, A349V, F508C;S1251N, H199Y, L1480P, R334Q, S1251N, A455E, H939R, M152V, R347H, S1255P, A554E, F575Y, H1054D, M265R, R347L, T338I, A1006E, F1016S, H1085P, M952I, R347P, T1036N, A1067T, F1052V, H1085R, M952T, R352Q, T1053I, D110E, F1074L, H1375P, M1101K, R352W, V201M, D110H, F1099L, I148T, P5L, R553Q, V232D, D192G, G27R, I175V, P67L, R668C, V456A, D443Y, G85E, I336K, P205S, R751L, V456F, D443Y;G576A;R668C, G126D, I502T, P574H, R792G, V562I, D579G, G178E, I601F, Q98R, R933G, V754M, D614G, G178R, I618T, Q237E, R1066H, V1153E, D836Y, G194R, I807M, Q237H, R1070Q, V1240G, D924N, G194V, I980K, Q359R, R1070W, V1293G, D979V, G314E, I1027T, Q1291R, R1162L, W361R, D1152H, G463V, I1139V, R31L, R1283M, W1098C, D1270N, G480C, I1269N, R74Q, R1283S, W1282R, E56K, G551D, I1366N, R74W, S13F, Y109N, E60K, G551S, K1060T, R74W;D1270N, S341P, Y161D, E92K, G576A, L15P, R74W;V201M, S364P, Y161S, E116K, G576A;R668C, L165S, R74W;V201M;D1270N, S492F, Y563N, E193K, G622D, L206W, R75Q, S549N, Y1014C, E403D, G628R, L320V, R117C, S549R, Y1032C, E474K, G970D, L346P, R117G, S589N, E588V, G1061R, L453S, R117H, S737F*.

Although it is already possible to observe the great clinical impact of these drugs, there are still many economic, social, and clinical challenges to be faced to enable their large-scale use. One of the biggest obstacles is the cost of these medications, which are around €150–200,000 per patient per year, causing many countries to not fund the therapies through their health systems (30). Furthermore, it is important to remember that the therapeutic effects of CFTR modulators depend on the mutation and class to which they belong, which excludes a significant portion of the population with CF. Finally, there is a considerable risk that the use of CFTR modulators may cause drug-drug interactions in cytochrome P450 3A4 and various adverse effects (31). For these reasons, new compounds with different mechanisms of action are already in the clinical trial phases, one of them (32) already being in phase 3. In addition, therapies are currently being studied that aim to correct protein function through other pathways, such as those with mRNA and viral vectors. The mRNA therapy aims to restore the CFTR protein to adequate levels through a distribution of normally functioning encoded mRNA copies, regardless of the patient's mutation (30). Therapy with viral vectors consists of inserting complementary DNA with normal CFTR into plasmid DNA, aiming for a transfection in lung cells through viral vectors (30, 33).

The impact of these discoveries over the years is evident, achieving advances not only in the pulmonary function of patients, but also in its quality and life expectancy (Figure 1). However, several obstacles remain to be overcome for the development of new therapies, such as the inclusion of patients with rarer mutations, treatment costs, regulatory difficulties in carrying out large clinical trials, among others (13, 34–36).

### Modulators Impact on Life Expectancy: A New Era in CF Clinics

The CF Foundation Registry was created in 1966 to track the health of people with cystic fibrosis who receive care at CF Foundation-accredited care centers and agree to share their data to inform continued quality improvement in treatment and specialized care. Each year, the CF Foundation analyzes these data and shares this information with the CF community through the Patient Registry Annual Data Report. Based on 2019 Registry data, the life expectancy of people with CF who are born between 2015 and 2019 is predicted to be 46 years. Data also show that of the babies who are born in 2019, half are predicted to live to be 48 years or older (37). The latest CF Foundation Patient Registry data show steady gains in survival for people with CF.

In conclusion, advances in diagnosis and treatment, as well as improvements and expansion in multidisciplinary care centers, have changed the situation for CF patients, resulting in a significant increase in life expectancy. Expanding the use of new drugs may result in improved mortality rates, life expectancy, and quality of life for CF patients.

## Concluding Remarks

Therefore, we conclude that advances in basic science related with CF (genetics and proteomics) has been essential for the developments of recent achievements in the survival and quality of life in CF. Advances in CF are a real and unique example in translational medicine. It is essential to continue the efforts in the search for new treatment strategies for CF to minimize its effects over time, optimize treatment, increase the quality of life of patients, reduce inequalities in access between them, and extend their survival.

## Author Contributions

All authors listed have made a substantial, direct, and intellectual contribution to the work and approved it for publication.

## Funding

The present work was carried out with the support of the Coordination of Improvement of Personnel Higher Education - Brazil (CAPES) - Financing Code 001. The funders had no role in study design, data collection and analysis, decision to publish, or preparation of the manuscript.

## Conflict of Interest

The authors declare that the research was conducted in the absence of any commercial or financial relationships that could be construed as a potential conflict of interest.

## Publisher's Note

All claims expressed in this article are solely those of the authors and do not necessarily represent those of their affiliated organizations, or those of the publisher, the editors and the reviewers. Any product that may be evaluated in this article, or claim that may be made by its manufacturer, is not guaranteed or endorsed by the publisher.
